# Functional analysis of *GhCHS*, *GhANR* and *GhLAR* in colored fiber formation of *Gossypium hirsutum* L

**DOI:** 10.1186/s12870-019-2065-7

**Published:** 2019-10-29

**Authors:** Jianfang Gao, Li Shen, Jingli Yuan, Hongli Zheng, Quansheng Su, Weiguang Yang, Liqing Zhang, Vitalis Ekene Nnaemeka, Jie Sun, Liping Ke, Yuqiang Sun

**Affiliations:** 1 Plant Genomics & Molecular Improvement of Colored Fiber Lab, Key Laboratory of Plant Secondary Metabolism and Regulation of Zhejiang Province, College of Life Sciences and Medicine, Zhejiang Sci-Tech University, Hangzhou, 310016 Zhejiang China; 2 College of Agriculture/The Key Laboratory of Oasis Eco-Agriculture, Shihezi University, Shihezi 832000, Xinjiang, China

**Keywords:** *G. hirsutum* L., Fiber color, Anthocyanidin, *GhCHS*, *GhANR*, *GhLAR*

## Abstract

**Background:**

The formation of natural colored fibers mainly results from the accumulation of different anthocyanidins and their derivatives in the fibers of *Gossypium hirsutum* L. Chalcone synthase (CHS) is the first committed enzyme of flavonoid biosynthesis, and anthocyanidins are transported into fiber cells after biosynthesis mainly by Anthocyanidin reductase (ANR) and Leucoanthocyanidin reductase (LAR) to present diverse colors with distinct stability. The biochemical and molecular mechanism of pigment formation in natural colored cotton fiber is not clear.

**Results:**

The three key genes of *GhCHS*, *GhANR* and *GhLAR* were predominantly expressed in the developing fibers of colored cotton. In the *GhCHSi*, *GhANRi* and *GhLARi* transgenic cottons, the expression levels of *GhCHS*, *GhANR* and *GhLAR* significantly decreased in the developing cotton fiber, negatively correlated with the content of anthocyanidins and the color depth of cotton fiber. In colored cotton Zongxu1 (ZX1) and the *GhCHSi*, *GhANRi* and *GhLARi* transgenic lines of ZX1, HZ and ZH, the anthocyanidin contents of the leaves, cotton kernels, the mixture of fiber and seedcoat were all changed and positively correlated with the fiber color.

**Conclusion:**

The three genes of *GhCHS*, *GhANR* and *GhLAR* were predominantly expressed early in developing colored cotton fibers and identified to be a key genes of cotton fiber color formation. The expression levels of the three genes affected the anthocyanidin contents and fiber color depth. So the three genes played a crucial part in cotton fiber color formation and has important significant to improve natural colored cotton quality and create new colored cotton germplasm resources by genetic engineering.

## Background

Cotton, as one of the most important economic crops, provides more than 50% of the fiber source in the textile industry [1]. However, the printing and dyeing processes contain many carcinogens, resulted in bleaching difficulties, containing high concentrations of halides organic matter, most organic halides are carcinogenic, teratogenic and mutagenic [2–6] are also typical persistent organic pollutants [7, 8], which are very difficult to recover. Fortunately, the natural colored cotton fibers yarn without or very less dyeing directly into cloth and show great application prospects in the textile industry. Because of the green, ecological and eco-friendly characteristics, the natural colored cotton and its fabrics are praised as “21^st^ century Darling”, and also undoubtedly becoming an important choice and way for the transformation and upgrading of the textile industry in China. But currently only two types of colored cotton with brown and green color are available in the actual production and textile industry, which seriously restricts the development of colored cotton industry [8–15]. Natural colored cotton fibers were pigmented by synthesizing and accumulating natural pigments in developing fiber, the previous reports showed the brown coloration of cotton fibers resulted from flavonoids synthesis and accumulation in fibers [3, 16], proanthocaynidins (PAs) biosynthesis and accumulation played the leading role for the brown coloration in cotton fibers [10, 11, 17–21]. Because of the complex pigment composition and structure in colored cotton fibers, it is difficult to genetically improve cotton fiber color, and the mechanism of pigment formation in the colored fiber is still unclear.

Flavonoids are one of the largest groups of secondary metabolites and widely distributed in plants ranging from spermatophytes to mosses [22–24]. The main classes of these phenylpropanoid pathway derivatives include flavonols, anthocyanins, and PAs. As water-soluble natural pigments, anthocyanins are responsible for the red, purple and blue colors of many flowers and fruit that attract pollinators and seed dispersers [25]. Over 600 anthocyanins have been identified in nature, the most common anthocyanins are pelargonidin, cyanidin, delphinidin, peonidin, petunidin, malvidin, and the derivatives of six widespread anthocyanidins, which constitute the dominant core anthocyanins in higher plants and exhibit different colors such as orange, red, purple, and blue in plant flowers, seeds, and fruits [26–29]. Plant flowers and fruits have a variety of colors because they are closely related to anthocyanins. The PAs play an important role in regulating many biological stresses and abiotic stresses in plant, and play a crucial part in the physiological processes such as anti-ultraviolet, disease resistance, scavenging free radicals, regulating seed dormancy and germination [29–34]. The anthocyanin biosynthetic pathway is an important branch of the general flavonoid pathway, the chalcone synthase (CHS) is the first key regulatory enzyme which mediated synthesis of naringenin chalcone from 4-coumaroyl-CoA and malonyl-CoA. After biosynthesis, flavonoids are transported to vacuoles or cell walls [35]. The last steps of monomer biosynthesis are catalyzed by two distinct enzymes of leucoanthocyanidin reductase (LAR) and anthocyanidin reductase (ANR). LAR directly reduced leucoanthocyanidins to the corresponding 2, 3-trans-(6)-flavan-3-ols such as catechin as the first step in PA biosynthesis. The anthocyanidin synthase (ANS) converted leucoanthocyanidins into the 2, 3-cis-type anthocyanidins such as epicatechin, and then anthocyanidins were reduced by ANR to synthesis the corresponding 2, 3-cis-flavan-3-ol [36]. The anthocyanin branch point enzyme UDP-glycose: flavonoid-3-O-glycosyltransferase (UF3GT) and the PA branch point enzyme ANR both utilize the unstable flavonoid precursor cyanidin as a substrate. LAR also converted 4b-(S-cysteinyl)-epicatechin into free epicatechin for regulating the length of PA polymers in *Medicago truncatula* [37]. Both LAR and ANR are NADPH/NADH-dependent isoflavone-like reductases belonging to the reductase epimerase-dehydrogenase superfamily.

So engineering paler color has been achieved relatively easily by silencing structural genes in the anthocyanin biosynthetic pathway. Shifts in color from blue to red have been achieved by silencing Flavanone 3, 5-Hydroxylase gene (*F3’5’H*) [38]. Novel, red colored seeds of soybean have been produced by inhibiting the activity of ANR in the seed coat [39]. The significantly suppressed soybean *ANR1* and *ANR2* genes resulted in the red-brown grain phenotype, the effects of silencing *ANR1* and *ANR2* redirected metabolic flux from PA biosynthesis into the anthocyanin and flavonol-3-*O*-glucoside pathways. ANR removed anthocyanidins to supply epicatechin for proanthocyanidin synthesis. In the absence of ANR activity, red cyanidin-based anthocyanins accumulated in the seed coat. LAR and ANR are the key enzymes of anthocyanin transport and proanthocyanidin synthesis.

The PA branchpoint genes in soybean seed coat tissue were silenced as a novel methods for genetically improved pigmentation in plants. Many economical crops also accumulated PAs in the seed coats, *ANR* gene could be suppressed for genetically modified grain color in canola (*Brassica napus* L.), flax (*Linum usitatissimum*), and wheat (*Triticum* spp.) [40–42].

In this study, the three key genes for anthocyanidin biosynthesis and transport in natural colored cotton fiber were analyzed. The fiber color was altered with the decreased transcript level of three key genes, which resulted in the content of anthocyanidins change. It is very important to genetic manipulation *GhCHS*, *GhANR* and *GhLAR* in the anthocyanin metabolic pathway to improve the cotton fiber color, to satisfy the increasing great demand for green textile industry.

## Results

### Identification and phylogenetic analysis of *GhCHS*, *GhLAR* and *GhANR* genes

**Fig. 1:**
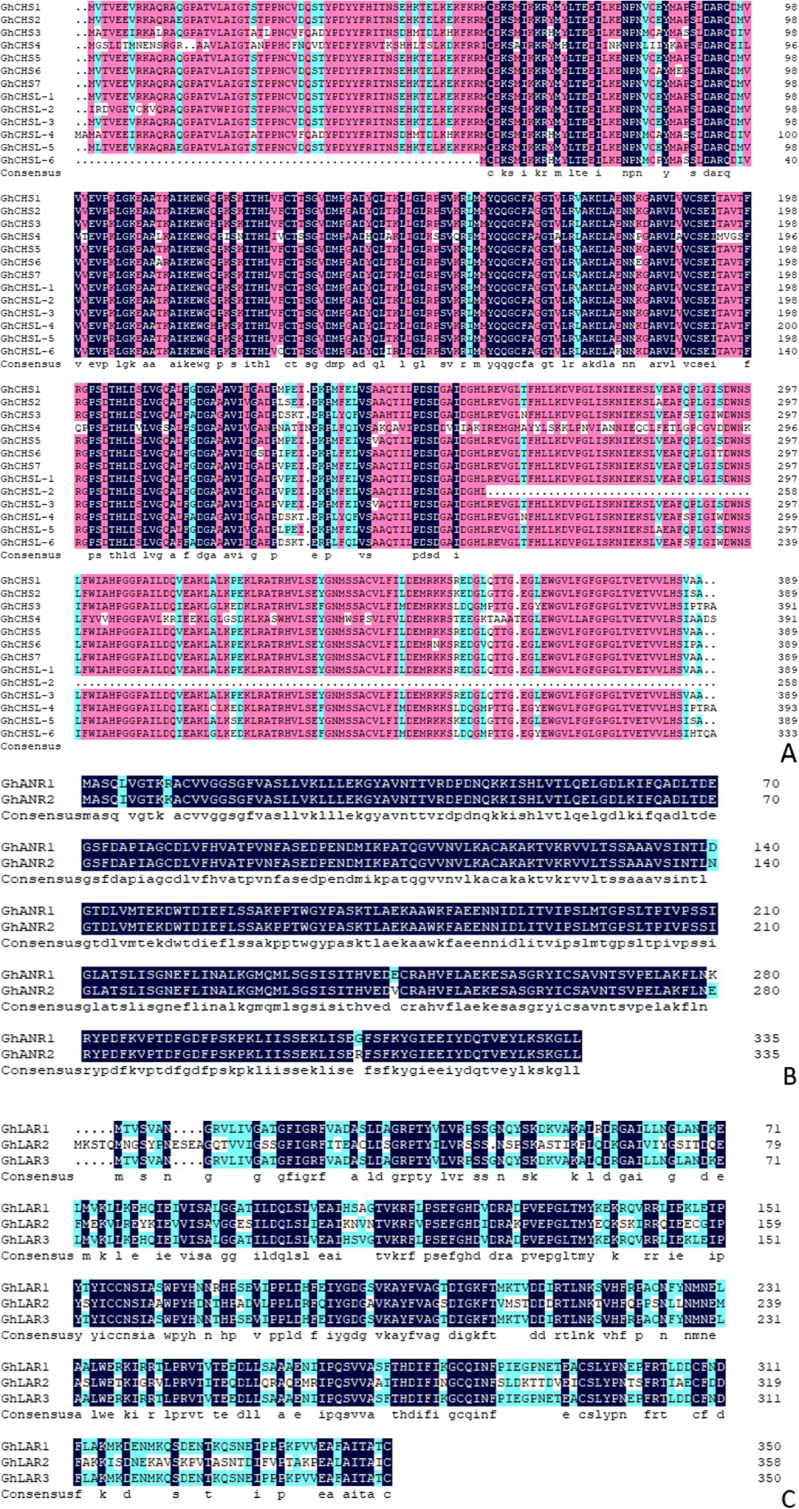
Clustal W alignment of multiple amino acid sequences alignment of GhCHS, GhANR and GhLAR. Colors indicate the similarity of amino acids sequences coded by the GhCHS (**a**), GhANR (**b**), GhLAR (**c**)

The differentially expressed genes were scanned from the transcriptome of brown cotton and its near isogene line [11]. From the differentially expressed genes, the genes in the anthocyanidin biosynthesis pathway including *GhCHS*, *GhLAR* and *GhANR* were selected for further analysis in the colored fiber in *G. hirsutum*. In *G. hirsutum*, 7 *GhCHS* genes and 6 *GhCHS-like* genes were scanned, 2 *GhANR* genes and 3 *GhLAR* genes were obtained (Fig. 1).

**Fig. 2:**
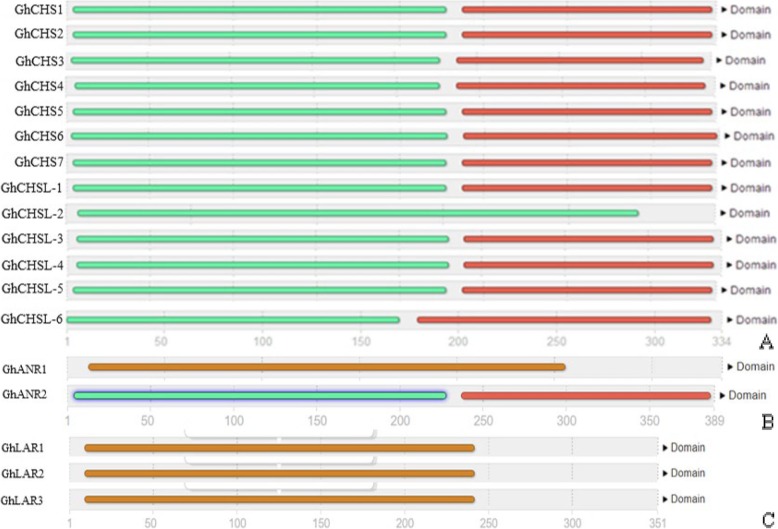
Structural and motif analysis of GhCHS, GhANR and GhLAR based on InterPro test

Multiple ChCHSs contained high amino acid homology, the homology of special motifs reached 100%, the *GhCHS* genes kept highly conserved in *G. hirsutum* (Fig. 1a). The *GhLAR* genes and *GhANR* genes were also much conserved in *G. hirsutum* (Fig. 1b, c). The members in the GhCHS family except GhCHSL-2 had two domains and were mostly divided into N-terminus and C-terminus, but GhCHSL-2 has only N-terminal one (Fig. 2a). The GhANR1 and GhANR2 were also divided into N-terminal and C-terminal (Fig. 2b). GhLARs had only one N-terminal domain (Fig. 2c).

### Expression pattern of *GhCHS*, *GhLAR* and *GhANR* in the developing fibers

**Fig. 3:**
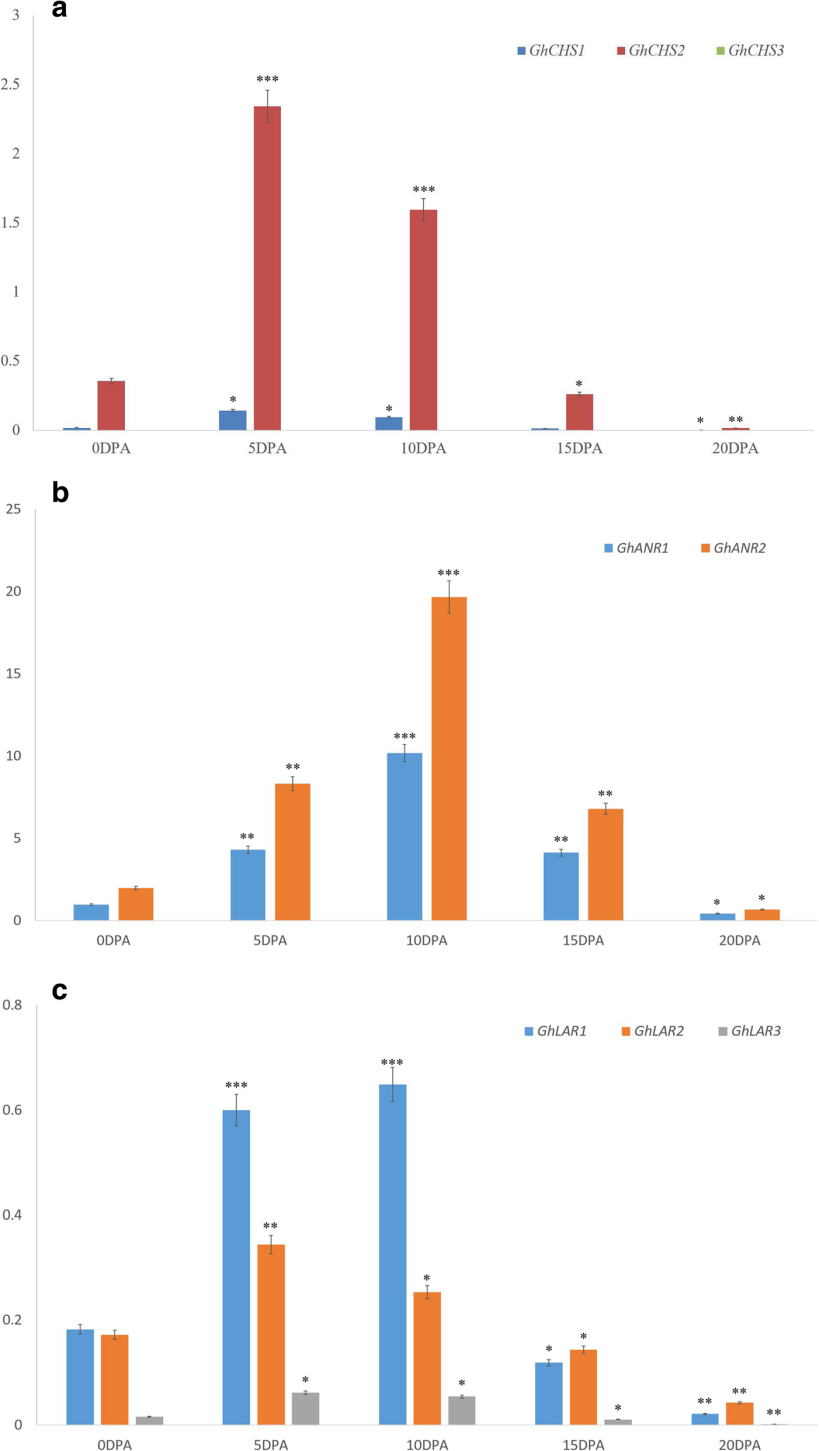
The expression analysis of *GhCHS*s, *GhLAR*s and *GhANRs* in the developing fiber of 0, 5, 10, 15 and 20 DPA in natural brown cotton ZX1. Data presented in all graphs are means ± SD (*n* = 3). (Student’s t-test, **P < 0.05, **P < 0.01, ***P < 0.005, compared to 0 DPA)

The 7 *GhCHS* genes and 6 *GhCHS-like* genes, 2 *GhANR* genes and 3 *GhLAR* transcript levels in the developing fibers of different stages in the natural colored cotton Zongxu1 (ZX1) and different cotton species were measured. The 3 *GhCHS* genes (named *GhCHS1*, *GhCHS2*, and *GhCHS3*) were detected in the developing fibers of ZX1, and *GhCHS2* were predominant. The expression level of *GhCHS2* was extremely higher than *GhCHS1* and *GhCHS3* and appeared most especially in the fiber of 5 and 10 DPA (days post anthesis) (Fig. 3a). The two *GhANR* genes (*GhANR1* and *GhANR2*) were quantified in the developing fibers of ZX1, the maximal expression level appeared in the fiber of 10 DPA (Fig. 3b). The expression levels of *GhANR* genes were extremely higher than those of *GhLAR* genes to about 10-fold in the fibers of 5 DPA and 30-fold in the fibers of 10 DPA. All *GhLAR* genes were detected in the developing fibers from 0 DPA to 20 DPA, predominantly expressed in the developing fibers of 5 DPA and 10 DPA (Fig. 3c). From the expression pattern of *GhCHS*, *GhLAR* and *GhANR*, the 3 genes were all predominantly expressed in the fibers of 10 DPA, the gene expression patterns were further detected in different cotton species with different colored fibers at 10 DPA.

**Fig. 4:**
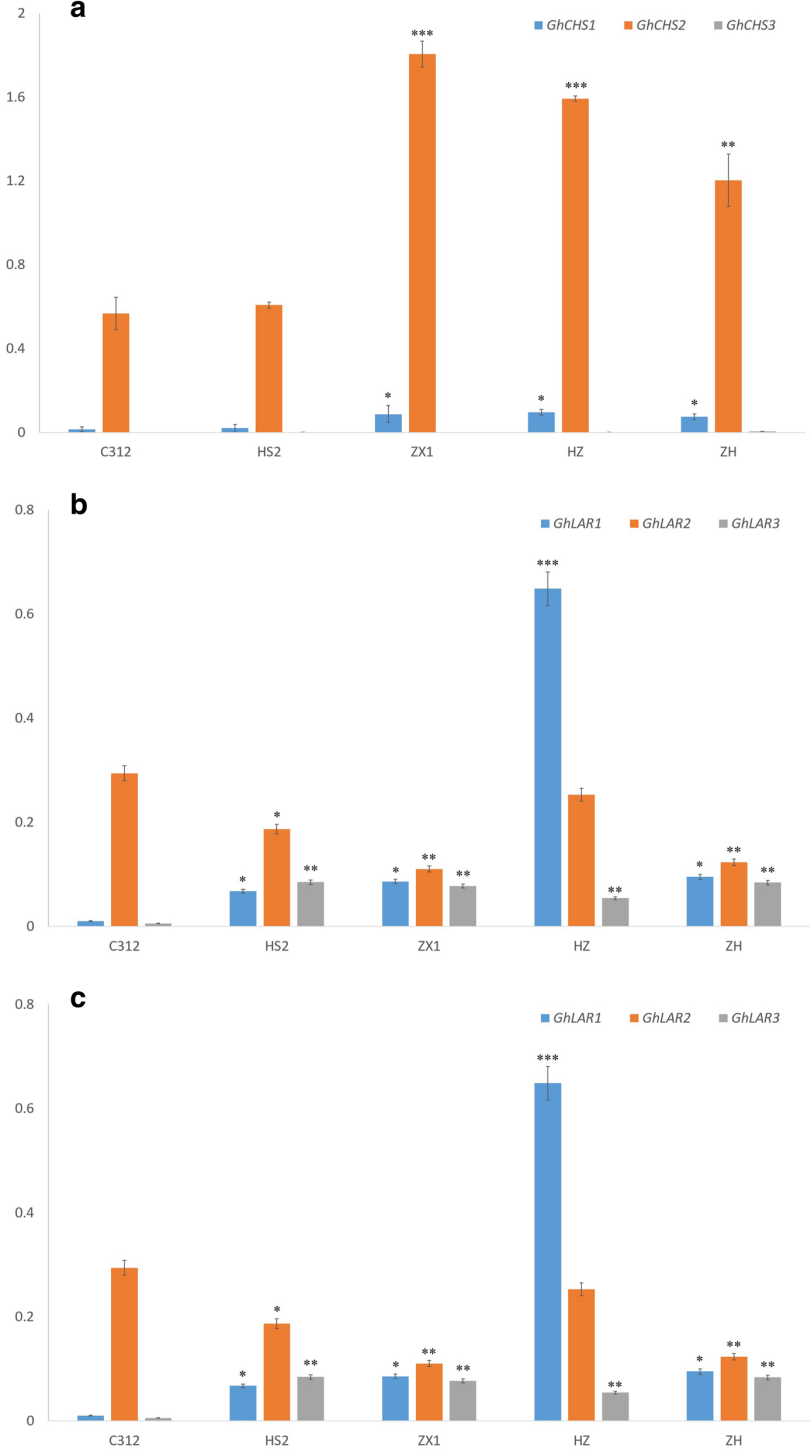
The expression analysis of *GhCHS*, *GhLAR* and *GhANR* genes in the developing fiber of 10 DPA in 5 cotton species. Data presented in all graphs are means ± SD (*n* = 3). (Student’s t-test, *P < 0.05, **P < 0.01, ***P < 0.005, compared to C312)

The 5 cotton species (with white fiber or brown fiber) were used to measure *GhCHS*, *GhLAR* and *GhANR* expression levels in the developing fibers of 10 DPA. In plant, *chalcone synthase* (*CHS*) gene is the first committed step of flavonoid biosynthesis, the expression levels of *GhCHS* genes were significantly higher in the colored fibers than in the white fibers, especially in ZX1 and HZ fibers of 10 DPA (Fig. 4a). For the *GhCHS* genes*, GhCHS2* kept preferential expression and maintained the trend during the colored fiber development, *GhCHS1* was weakly expressed and the expression level of *GhCHS3* was almost negligible, so the *GhCHS2* was measured to represent the transcript levels of *GhCHS* genes in the developing fibers, and the conserved sequence was used to interfere their transcripts. The expression levels *GhANR* genes in colored cotton HZ lines with dark brown fiber were the highest among the 5 cotton species. The expression of *GhANR2* was greatly increased in the dark brown fibers of HZ compared with that in C312, HS2, ZH and ZX1; the transcript level of *GhANR1* in ZH lines with light brown was significantly higher than that in C312, HS2, and ZX1 (Fig. 4b). For anthocyanidins transport, the ANR represents the main way for anthocyanidins flowing into fiber cell in natural colored cotton, the expression level of *GhANR* genes in the developing fiber of the 5 species was extremely higher than that of *GhLAR* genes and *GhCHS* genes (Fig. 4). Compared with white cotton fibers, the transcription level of *GhANR* genes in brown cotton fibers was significantly higher than in white fibers. *GhLAR1* gene had the highest expression levels in the deep brown fibers of HZ lines among the 5 cotton species (Fig. 4c), significantly higher than the natural colored cotton ZX1, ZH and white fiber cotton C312 and HS2. So the conserved sequences of *GhANR1* and *GhANR2*, *GhLAR1* were used to interfere their transcripts.

### Phenotypic analysis of transgenic RNAi colored cotton

**Fig. 5:**
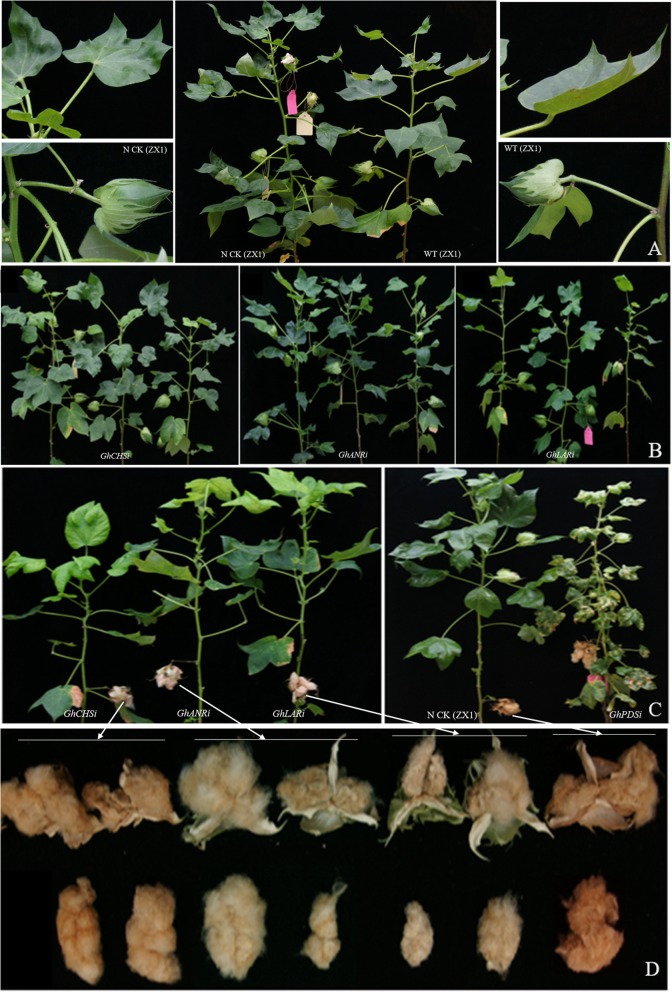
Phenotypic analysis of plant, boll and fiber in *GhCHS*-RNAi (*GhCHSi*), *GhANR*-RNAi (*GhANRi*), *GhANR*-RNAi (*GhANRi*), *GhPDS*-RNAi (*GhPDSi*), negative controls (N CK) of ZX1 and wild cotton of ZX1 plants (WT ZX1). **a**: the phenotype of negative controls ZX1 (N CK) and wild cotton ZX1 plants (WT ZX1). **b**: the transgenic plants of *GhCHSi*, *GhANRi*, *GhANRi*. **c**: the transgenic plants of *GhCHSi*, *GhANRi*, *GhANRi* with opening bolls. **d**: the phenotype of boll and fiber of *GhCHSi*, *GhANRi*, *GhANRi* plants

**Fig. 6:**
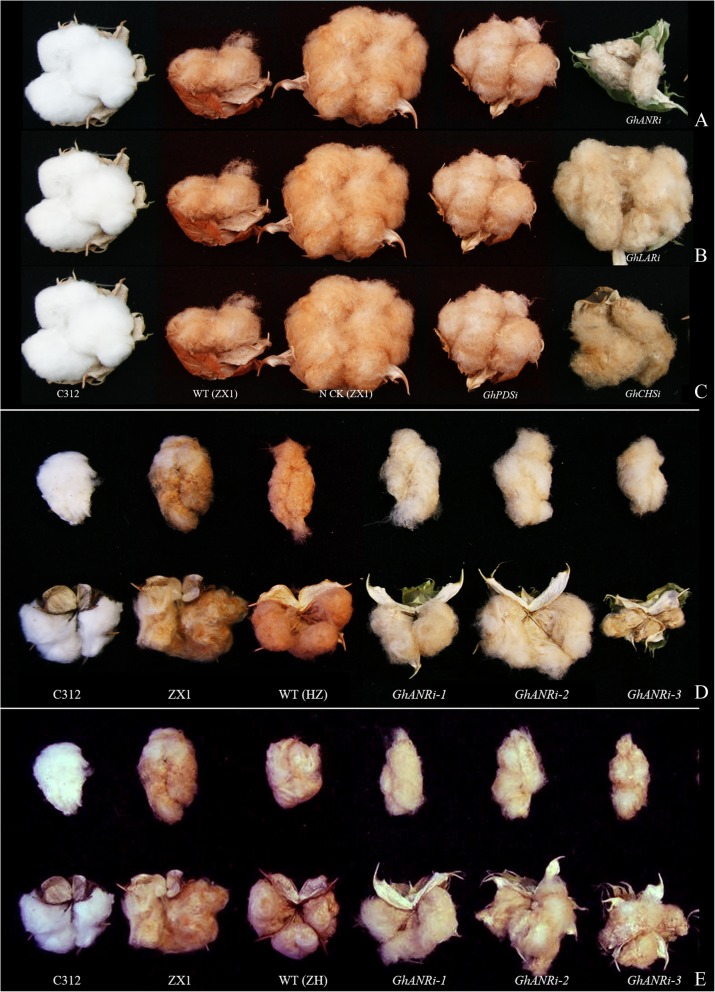
The phenotypic comparison of boll and fiber in *GhCHSi*, *GhANRi*, *GhANRi*, *GhPDSi*, and different controls of white fiber C312, donor cotton of natural colored cotton (ZX1, HZ and ZH) and C312. **a**: the phenotypic comparison of fiber in transgenic ZX1 lines of *GhCHSi* (**a**), *GhANRi* (**b**), *GhANRi* (**c**), *GhPDSi* (positive control ZX1) and different controls of white fiber C312, ZX1, negative control ZX1 (N CK, transgenic ZX1 with vector-free). **d**: the phenotypic comparison of fiber in transgenic HZ lines of *GhCHSi*, *GhANRi*, *GhANRi*, and different controls (HZ with dark brown fiber). **e**: the phenotypic comparison of fiber in transgenic ZH lines of *GhCHSi*, *GhANRi*, *GhANRi*, and different controls (ZH with light brown fiber)

The natural colored cotton ZX1 was used to silence the endogenous *GhCHS2*, *GhLAR1* and *GhANR* genes through CLCrV-based virus-induced gene silencing system. The positive control of transgenic *GhPDS*-RNAi plant appeared light bleaching symptoms in the leaves, stalks, cotton bolls and cotton fiber, which continued to be expressed in the whole life of cotton. The negative control of transgenic vector-free plants (ZX1 NCK) compared with the wild type only showed the shrinkage of the leaves (Fig. 5). The color of fibers in the *GhCHSi*, *GhANRi* and *GhLARi* were obviously fading in the depth of brown color (Figs. 5, 6). The fiber color in *GhANRi* plants was distinctly faded with brown color and significantly lighted compared to ZX1 (N CK), the fiber color in *GhLARi* plants became lighter in the depth of brown color, the cotton fiber color in *GhCHSi* plants was not significantly different from ZX1, *GhPDSi* and its N CK (Fig. 6a,b,c). Among the 5 cotton species, the fiber color of HZ was deeper than the other 4 cotton species, the fiber color of *GhANRi* HZ plants was obviously lighter than that of WT (HZ) and ZX1 (Fig. 6d). The fiber color in *GhANRi* ZH plants also obviously became lighter than WT (ZH) and ZX1 (Fig. 6e). It indicated that *GhANR* and *GhLAR* played an important role in the anthocyanin synthesis and the accumulation of pigment in cotton fiber.

### Expression analysis of *GhCHS*, *GhANR* and *GhLAR* in RNAi plants

**Fig. 7:**
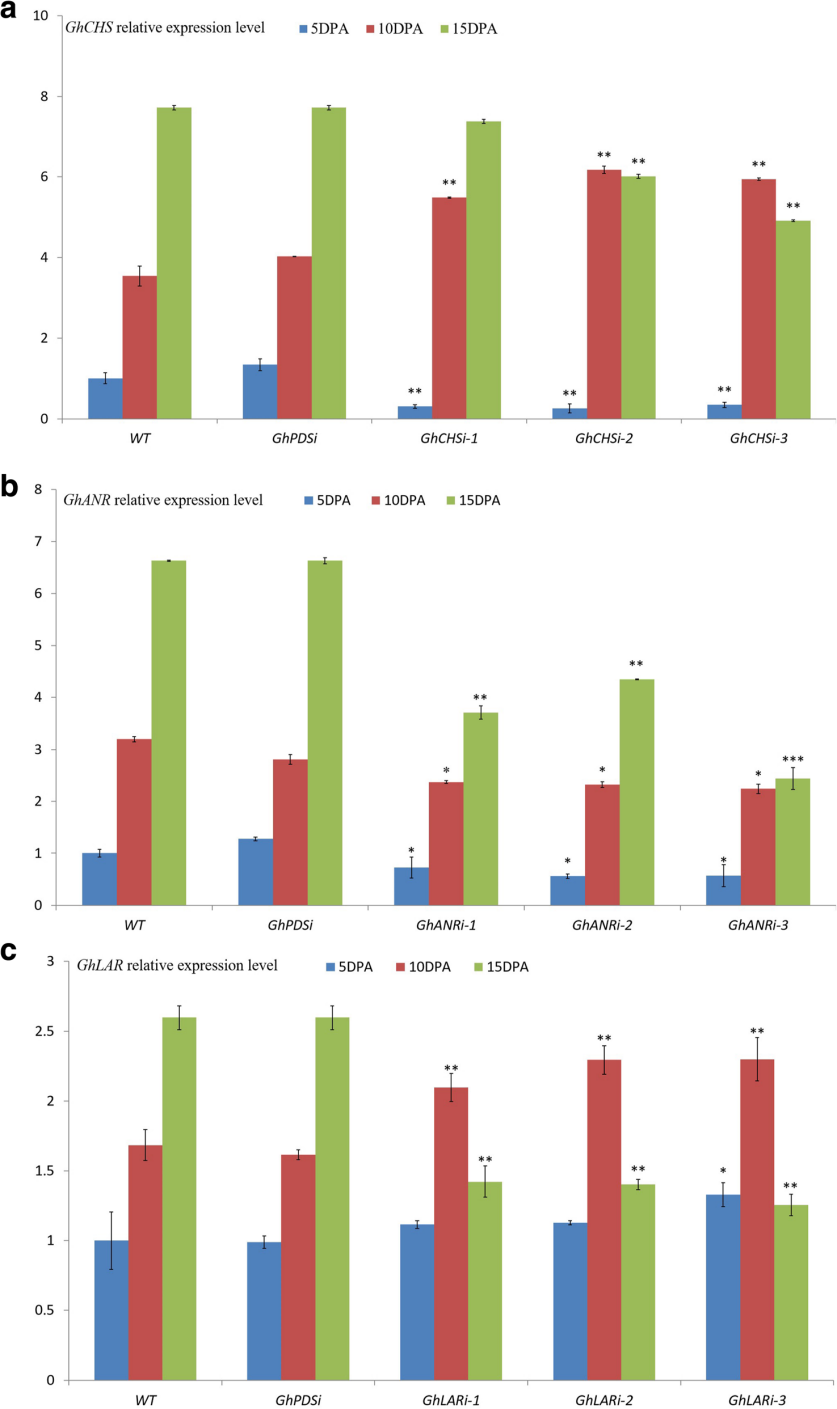
Relative expression levels of *GhCHS*, *GhANR*, *GhANR* in the developing fiber of 5 DPA, 10 DPA and 15 DPA in *GhCHSi*, *GhANRi*, *GhANRi*, *GhPDSi* transgenic cotton lines and WT ZX1. **a**: Relative expression analysis of *GhCHS* in *GhCHSi*, *GhPDSi* transgenic cotton lines and WT. **b**: Relative expression analysis of *GhANR* in *GhANRi*, *GhPDSi* transgenic cotton lines and WT. **c**: Relative expression analysis of *GhLAR* in *GhLARi*, *GhPDSi* transgenic cotton lines and WT. Data presented in all graphs are means ± SD (*n* = 3), (Student’s t-test, *P < 0.05, **P < 0.01, ***P < 0.005, compared to WT)

In the gene-silenced ZX1 plants, the expression levels of *GhANR*, *GhLAR* and *GhCHS* were significantly changed compared to the WT ZX1 and *GhPDSi* ZX1 plants (Fig. 7). In the *GhCHSi* ZX1 plants, the expression level of *GhCHS2* in the fibers at 5 DPA and 15 DPA was significantly lower than that of WT ZX1 and *GhPDSi* ZX1, especially in the developing fiber of 5 DPA (Fig. 7a). The expression level of *GhLAR* in the *GhCHSi* ZX1 plants appeared no significant change (Additional file 1: Figure S1A); the expression level of *GhANR* in the developing fiber of 15 DPA were significantly decreased (Additional file 1: Figure S1B). The brown color of fiber in *GhCHSi* ZX1 plants was lightly fading (Figs. 5, 6). In the *GhANRi* ZX1 plants, the *GhANR* expression level in the developing fibers of 5 DPA, 10 DPA and15DPA were significantly lower than WT ZX1 and *GhPDSi* ZX1 (Fig. 7b), the expression level of *GhCHS* in the fibers of 15 DPA increased, but had no significant change in the fibers of 5 DPA and 10DPA (Additional file 1: Figure S1C). The expression level of *GhLAR* had various changes (Additional file 1: Figure S1D). The color of brown fiber in *GhANRi* ZX1 plants was strongly fading (Figs. 5, 6). Compared with WT ZX1 and *GhPDSi* ZX1 plants, the expression level of *GhLAR* in the fiber of 5 DPA in the *GhLARi* plants was not all significantly changed, but was markedly increased in the fiber at 10 DPA, sharply decreased in the fibers at 15 DPA (Fig. 7c), the expression level of *GhCHS* in the fibers of 15 DPA was significantly increased (Additional file 1: Figure S1E), the expression level of *GhANR* in the fiber of 15 DPA was significantly increased in *GhLARi*-1 plant (Additional file 1: Figure S1F). The color of brown fiber in *GhLARi* ZX1 plants was significantly fading (Figs. 5, 6). From the *GhANRi* and *GhLARi* ZX1 plants, the suppression of *GhANR* and *GhLAR* could upregulate the expression of *GhCHS* gene (Additional file 1: Figure S1).

### The anthocyanin content in plant tissues positively correlated with fiber color

**Fig. 8:**
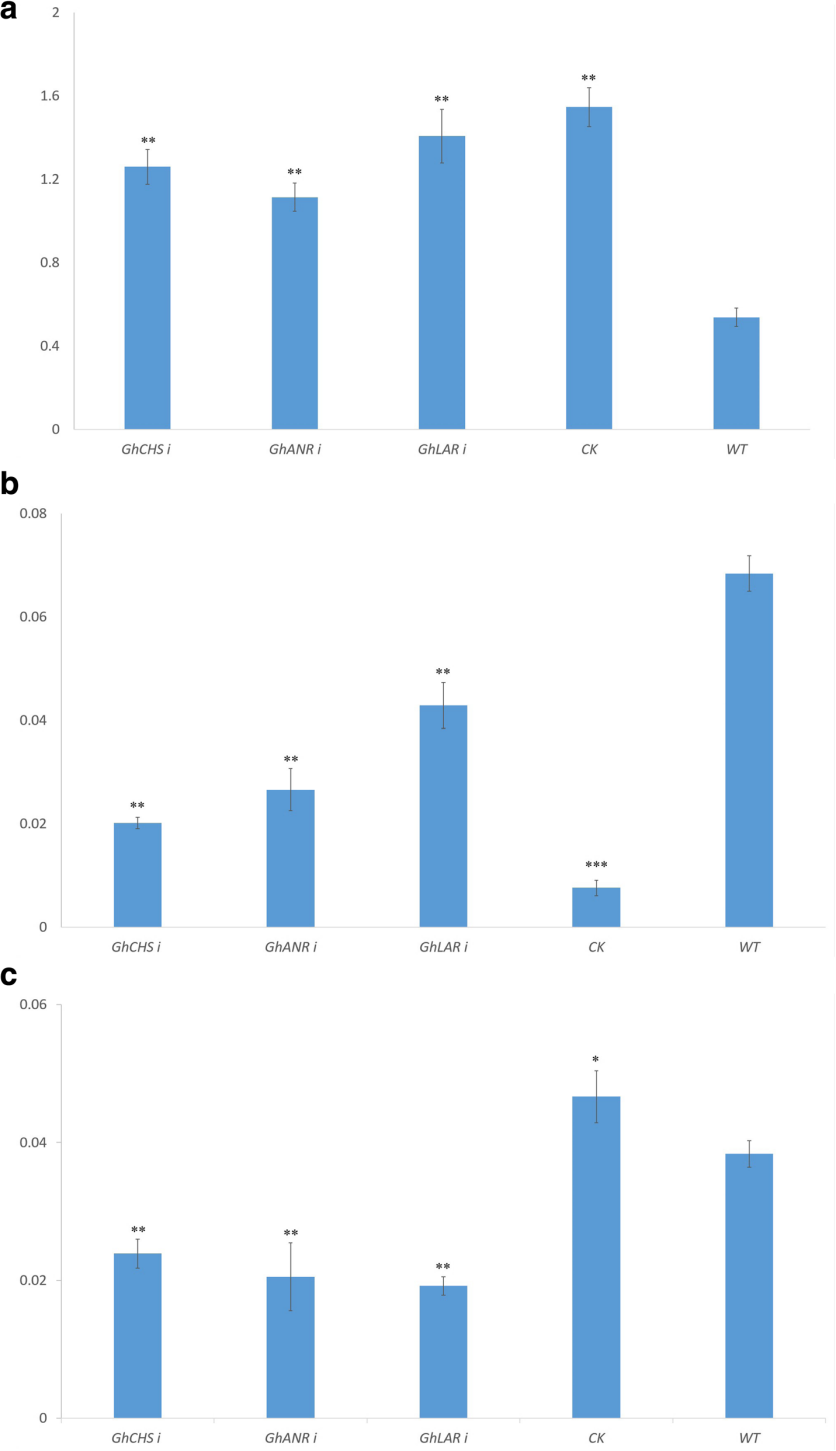
The anthocyanidins content in transgenic plants of *GhANRi*, *GhLARi* and *GhCHSi*, ZX1 (WT) and N CK ZX1. **a**: The anthocyanidins content in leaves of *GhANRi*, *GhLARi* and *GhCHSi* plants, ZX1 and N CK (Transgenic ZX1 with vector-free); **b**: The anthocyanidins content in cotton kernels of *GhANRi*, *GhLARi* and *GhCHSi* plants, WT and N CK; C: The anthocyanidins content in fiber and seedcoat in *GhANRi*, *GhLARi* and *GhCHSi* plants, ZX1 and N CK

Natural colored cotton ZX1 was large-area planted with brown fiber, here was used to study the effect of *GhANR*, *GhLAR* and *GhCHS* expression on anthocyanidins accumulation. The content of anthocyanidins of cotton kernel, fiber and seedcoat in WT ZX1 was significantly higher than those in the *GhANRi*, *GhLARi* and *GhCHSi* ZX1 plants (Fig. 8b, c). The contents of anthocyanidins in the RNAi plants were markedly decreased in the cotton kernels, fiber and seedcoat, but the anthocyanidins content was significantly increased in the leaves compared to those in WT ZX1 (Fig. 8a). The contents of anthocyanidins in leaves and cotton kernels of control plants with free-armed vector (N CK) were significantly higher than those in WT (Fig. 8a, c). The content of anthocyanidins in cotton kernels, fiber and seedcoat influenced the fiber color, the fiber color became fading with the anthocyanidins contents reduced in the *GhANRi*, *GhLARi* and *GhCHSi* plants, while the content of anthocyanidins in leaves were increased (Fig. 8b, c).

## Discussion

### Identification and expression pattern of *GhCHS*, *GhANR* and *GhLAR*

In the genome of *G. hirsutum*, the 13 *GhCHS* and *GhCHS-like* genes in the *CHS* family, 2 *GhANR* genes and 3 *GhLAR* genes were scanned, the gene and protein sequences of *GhCHS*, *GhANR* and *GhLAR* were highly conserved, but the genes of *GhCHS*, *GhANR* and *GhLAR* had the expression specificity in cotton plant, *GhCHS2* gene was predominantly expressed in colored cotton fibers, *GhCHS1* and *GhCHS3* expressed weakly in the developing fibers, the other *GhCHS* and *GhCHS-like* transcripts in the developing fibers were not measured. *GhLAR1*, *GhLAR2* and *GhLAR3* were all expressed in the developing fibers, but differentially expressed in the different cotton species with different colors or color-depth, the 3 *GhLAR* genes represented the high expressive abundance in the deeply colored fibers of HZ, and perhaps the *GhLAR* genes could improve the fiber color depth. The 2 *GhANR* genes were expressed in the developing fibers and obviously increased their transcripts in the colored cotton species, and also showed high expression abundance in the deeply colored fibers of HZ. Among the three types of genes for anthocyanidin biosynthesis and transport, the *GhANR* genes always maintained high expression level and as well represented the main flow way for anthocyanidins into fiber cell [11] and played the major role for anthocyanidins transport.

### The expression levels of *GhCHS*, *GhANR* and *GhLAR* closely related to fiber color

The 5 cotton species were used to measure the influence of *GhCHS*, *GhANR* and *GhLAR* gene expression on the fiber color formation. The expression levels of *GhCHSs* and *GhANRs*, *GhLAR1* and *GhLAR3* were all predominantly expressed early in developing fibers of colored fibers, especially in the dark brown fiber of HZ (Fig. 4). The expression levels of *GhCHS*, *GhANR* and *GhLAR* positively influenced the color formation of fiber in colored cotton. Therefore, for improving the color of cotton fiber, firstly the *GhCHS* gene expression would be increased to enhance the anthocyanin biosynthesis, then the *GhANR* and *GhLAR* increased their expression for transporting anthocyanidins into fiber cell. In the *GhANRi* and *GhLARi* cotton lines, the *GhCHS* gene was upregulated by the suppression of *GhANRi* and *GhLARi*, perhaps in natural colored cotton, the PA formation in the fiber cell could feedback the anthocyanidins biosynthesis, PA formation in fiber cell was mainly resulted from the anthocyanidin transport and accumulation through GhANR and GhLAR. Correspondingly, the suppression of *GhCHS* in *GhCHSi* cotton lines, the *GhANR* was downregulated, perhaps no more anthocyanidins could be transported into fiber cell through GhANR. The content of anthocaynidins in cotton kernels, fiber and seedcoat of *GhANRi*, *GhLARi* and *GhCHSi* plants decreased and increased pattern in leaves could confirm this hypothesis.

### The suppression of *GhCHS*, *GhANR* and *GhLAR* had negative effect on fiber color

The *GhANR*, *GhLAR* and *GhCHS* genes in natural colored cotton ZX1 was silenced, and the fiber color in the transgenic RNAi ZX1 plants was significantly different from the WT and CK. In the transgenic ZX1 plants, the endogenous genes of *GhANR*, *GhLAR* and *GhCHS* were suppressed, especially in the fiber of 5 DPA and 10 DPA (Fig. 7), the fiber color in the transgenic ZX1 plants faded to lighter and even more lighter. The down-regulation levels of the 3 genes emerged as negative correlation with fiber color. In the general phenylpropanoid pathway, chalcone synthase was the first committed enzyme of flavonoid biosynthesis, among the 3 genes, the conserved sequence of *GhCHS1, GhCHS2* and *GhCHS3* silenced has little significant effect on cotton fiber color. Firstly, it may be multiple members of *CHS* family in *G. hirsutum*, although *GhCHS2* predominantly expressed early in developing fiber in colored cotton, other members existed functional complementarity after *GhCHS2*, even *GhCHS1* and *GhCHS3* suppressed; Secondly, *GhCHS* genes were in the upstream location of anthocyanidin biosynthesis, suppression of *GhCHS* had little effect on downstream synthesis and metabolism of anthocyanins. In the early stage of anthocyanidin biosynthetic, *CHS*, *CHI*, and *F3H* are the common flavonoid pathway genes (also called early biosynthesis genes, EBGs), and are responsible for the biosynthesis of all downstream flavonoids. The varied expression profile of EBGs was not directly resulted in the change of anthocyanin content in *Solanaceous* vegetables [43]. The transcript of *SmCHS* was significantly increased in black or violet eggplant fruits compared to the light colored mutants of green or white [44, 45]. In potato tubers, *CHS* were highly expressed in red and purple tubers and more correlated with anthocyanin content [46–49].

The *GhANR* and *GhLAR* worked for anthocyanidins transport in the anthocyanin metabolic pathway, the *GhANR* played the main role for colored anthocyanidins into fiber cell, the *GhLAR* worked for transporting leucoanthocyanidin in fiber cell and also could enhance the fiber color perhaps by polymerization and oxidation to form anthocyanin derivatives [11]. The *GhLARs* were preferentially expressed in the deep colored fiber of HZ plant, the fiber color became lighter in the *GhLAR* suppressed plants.

PAs (also called condensed tannins) are synthesized via a branch of anthocyanin biosynthesis pathway under the catalyzation of LAR and ANR. LAR catalyzes the conversion of leucoanthocyanidin (flavan-3, 4-diol) to catechin, while ANR catalyzes the synthesis of epicatechin from anthocyanidin [36, 50, 51]. The tea *CsLAR* gene ectopically expressed in tobacco caused the accumulation of higher level of epicatechin than that of catechin, indicating LAR may be response for the biosynthesis of epicatechin [52]. ANRs in grapevine and tea had the epimerase activity and thus could convert anthocyanidin to a mixture of epicatechin and catechin [52, 53]. The metabolic fluxes were successfully genetically modified in soybean, *Arabidopsis*, and *petunia* to redirect the biosynthesis of the isoflavone, the flavonoid, and the anthocyanin, by suppressing the corresponding branchpoint genes [54–56]. The overexpressed *Medicago truncatula ANR* gene in tobacco reduced anthocyanin pigmentation in the flower and elevated PA levels [51]. Perhaps ANR competes with UF3GT for the substrate anthocyanidin, suppression of *ANR* genes results in increasing anthocyanin accumulations.

During early seed development, the seed coat was precociously accumulated cyanic pigments in the *Arabidopsis ANR* (or *BAN*) knockout mutant [57]. The pigments in seed coat was transitorily accumulated as a transparent *testa* (*tt*) phenotype and black pigmentation confined to the raphe of the dried grain [57]. This was different with the phenotype in soybean with red-brown grain by strongly suppressed *ANR* genes [39]. The underlying mechanistic and metabolite resulted in the different grain phenotypes of different species. In *Arabidopsis*, the *UF3GT* (*UGT78D2*) for anthocyanins in the seedling and the *ANR* for PAs in the seed coat were regulated reciprocally [58]. In soybean, *UF3GT* genes including *UGT78K1* and *UGT78K2*, ANR genes including *ANR1* and *ANR2* were expressed in the seed coat concurrently [39]. The difference of soybean grains phenotype perhaps resulted from *ANR* gene significant suppression. The presence and absence of *UF3GT* expression gave the *Arabidopsis ANR* knockout grain. Thus the red-brown grain phenotype in soybean was resulted from the accumulated of stable anthocyanins.

Two distinct enzymes of LAR and ANR were responsible for catalyzing the last step of the biosynthesis of flavan-3-ol monomers in PA-producing plants [52, 59, 60]. *LAR* and *ANR* gene occurs as single gene in *Arabidopsis* [36], or as multigene families, in grapevine [59] and tea [52]. Analysis of the *P. trichocarpa* genome revealed three loci encoding LAR and two loci encoding ANR [61, 62], which were likely involved in the catalysis of the last steps of flavan-3-ol biosynthesis in native black poplar from the enzymatic activity and in vitro enzyme assays. ANRs and LARs belong to two distinct classes of enzymes even with the similar evolutionary relationships, DFR was more related to ANRs than LARs [52, 62]. Freely available monomeric catechin was synthesized from the LAR branch and accumulated in black poplar, the concentration of free ANR-dependent epicatechin was very low. The epicatechin might contribute to the extension of PA chains in poplar, grape and Norway spruce [59, 63]. LARs promoted the biosynthesis of catechin monomers and inhibited their polymerization. The accumulation of catechin monomers and polymers was increased by up-regulating the expression of *NtLAR* and *NtANR* s in *CsMYB5b* transgenic tobacco [64]. So the transport of anthocyanidins through GhANR, GhLAR into fiber cell will be the important link for genetic engineering of colored fiber molecular improvement.

### The anthocyanidins content in the fiber directly influenced fiber color

In the transgenic *RNAi* cotton plants, the content of anthocyanidins was reduced by suppression of the endogenous *GhANR*, *GhLAR* and *GhCHS* genes, which resulted in the fiber color fading. CHS plays an important role in the phenylalanine metabolic pathway, plant growth and development, such as stress response, plant fertility and plant color [65]. LAR is a key enzyme in the synthetic pathway of plant flavonoids from phenylalanine, which catalyzes the conversion of colorless anthocyanins to catechins [51, 59, 60]. Transcript levels of LAR1 and ANR2 genes were significantly correlated with the contents of catechin and epicatechin to regulate PA synthesis, respectively. Ectopic expression of apple *MdLAR1* gene in tobacco suppresses expression of the late genes in anthocyanin biosynthetic pathway, resulting in loss of anthocyanin in flowers [60].

The anthocyanidins content in the fiber and seedcoat of *GhLARi* plants was higher than *GhANRi* plants, and the fiber color was also deeper than that of *GhANRi* plant, although LAR transported colorless anthocyanins into fiber cell. From our previous research, the transcription level of *GhLAR* in the fibers of brown cotton was higher than that in white cotton, during the fiber development, the fiber color of *GhLARi* plants lightly faded here. Compared with white cotton fibers, the expression level of *GhANR* in brown cotton fibers was significantly higher. The *GhANR* was actively expressed in brown cotton fibers and predominantly expressed at 12 DPA, when the transcript level of *GhANR* in brown cotton fibers was higher than that in white cotton fibers with 7-fold [11]. During the fiber development, the transcript level of *GhLAR* in brown cotton was much lower than that of *GhANR*, so effect of suppression of *GhLAR* on the fiber color change was lower than that of *GhANR*, the suppression of *GhANR* in ZX1 could cause the fiber color to be significantly lighter. The flavan-3-ols exist in brown and white cotton fibers as the 2, 3-cis form. The most of proanthocyanidins in brown fibers were prodelphidin (PD), while in white cotton fibers, PD content was similar to that of procyanidin (PC). The proanthocyanidin monomeric composition conformed with the expression profiles of proanthocyanidin synthase genes, and ANR played the key role in the proanthocyanidin biosynthesis in brown fibers. The proanthocyanidin synthase genes were expressed at a higher level in brown fibers than in white fibers [11]. The cis-form and trans-form of flavan-3-ols were synthesized in LAR and ANR branches, respectively [11, 36, 51, 66]. Mass spectrometry (MS) analyses revealed that the main PA monomers in brown cotton fibers contained three hydroxyls on the B ring (gallocatechin or epigallocatechin) [11, 21, 67], PA accumulation in brown fibers starts at an early stage (5 DPA) and peaks at 30 DPA, PAs are gradually converted into oxidized derivatives (quinones) in mature brown fibers. Because developing brown fibers do not exhibit distinct coloration until maturation, the condensed quinones maybe directly contribute to brown pigmentation in cotton fibers instead of their PA precursors [11]. Therefore, the three key genes in the anthocyanin metabolic pathways played the very important role in the coloration of cotton fibers, and became the target genes for genetic manipulation to improve cotton fiber color.

## Conclusions

In colored cotton fibers of *G. hirsutum*, *GhCHS2* gene was predominantly expressed in developing colored cotton fibers among 7 *GhCHS* and 6 *GhCHS-like* genes and represented *CHS* gene in anthocyanin metabolism in colored fibers. 2 *GhANR* genes and 3 *GhLAR* genes were highly conserved and homologous, significantly expressed in the developing colored cotton fibers. The *GhCHS2*, *GhANR* and *GhLAR* genes were differentially expressed in the colored cotton fibers with different color depth. The *GhCHS*, *GhANR* and *GhLAR* genes were interfered in colored cottons with different color depth, the expression levels of the three genes were significantly declined, the anthocyanin contents in the RNAi cotton plants were significantly reduced with the declined gene expression, and the fiber color was significantly changed and weaken. The three genes of *GhCHS*, *GhANR* and *GhLAR* played a crucial part in cotton fiber color formation and has important significant to improve natural colored cotton quality through genetic manipulation of the three genes and create new colored cotton germplasm resources by genetic engineering.

## Methods

### Plant materials

**Fig. 9:**
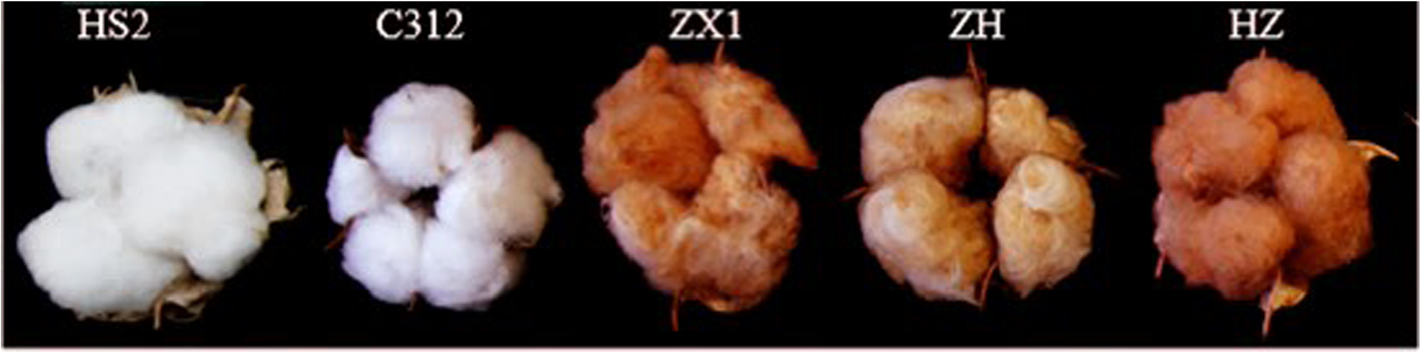
The phenotype of cotton bolls and fiber in HS2, C312, ZX1, ZH, HZ (*G. hirsutum* L.) used in the experiments

The *G. hirsutum* L. cv. Coker 312 (C312) and HS2 with white fiber, natural colored cotton ZX1 (Zongxu 1) with brown fiber, HZ with dark brown fiber and ZH with lighter brown fiber were used in this study (Fig. 9). The cotton seeds *G. hirsutum* cv. C312, HS2, ZH and HZ were preserved at the Key Laboratory for Plant Secondary Metabolism and Regulation of Zhejiang Province, Zhejiang Sci-Tech University, Hangzhou, China. C312 was an old commercial variety widely used for genetic transformation and provided by Mid-term National Cotton Germplasm Resource Bank (Institute of Cotton Research, CAAS). ZH, HZ and HS2 were new cotton lines cultivated by us. Cotton seeds of ZX1 were kindly provided by Dr. Xiongming Du (Institute of Cotton Research, CAAS). Seeds were germinated and grown in a greenhouse at 28 °C with a 14 h light and 10 h dark cycle. Seedlings with a 2nd true leaf emergence were used for agroinfiltration. Infiltrated plants were grown in the greenhouse at 23–25 °C with a 14/10 h light/dark photoperiod. The cotton plants were cultivated in the field under standard conditions. The samples of cottonseed, fiber and seedcoat were collected at the time of 0 DPA, 5 DPA, 10 DPA, 15 DPA and 20 DPA (days post anthesis) respectively (the fiber and seedcoat at 10, 15 and 20 DPA were removed from the cotton seed kernel), then put into liquid nitrogen and stored in the − 80 °C ultra-low temperature freezer for RNA extraction.

### Gene cloning and construction of RNAi vectors

The candidate genes were obtained from the differentially expressed genes in brown cotton and its near isogene line (*G. hirsutum*) [11]. The *GhANR*, *GhLAR* and *GhCHS* genes were scanned in the cotton genomes (http://www.cottongen.org) for gene accesses and sequences. BioEdit was used for multiple sequence alignment with the amino acid sequences of *GhANR1*, *GhANR2*, *GhLAR1*, *GhLAR2*, *GhLAR3*, and *GhCHS* genes. The characteristics of the genes coded proteins were used TMHMM (http://www.cbs.dtu.dk/services/TMHMM-2.0/) analysis for the transmembrane region of the protein encoded by mRNA. The components, physicochemical properties and isoelectric points of amino acid sequences are analyzed by ProtParam (http://web.expasy.org/ protparam/), respectively.

The new CLCrV-based vector was modified from the CLCrV DNA-A and DNA-B components individually, which were inserted into the pCambia1300 vector to generate pCLCrVA and pCLCrVB, respectively [68]. The fragments of candidate genes were inserted into pCLCrVA to produce pCLCrVA-*GhCHS*, pCLCrVA-*GhANR,* pCLCrVA-*GhLAR* for VIGS in cotton plants as described in the previous papers [68–70].

A 400–600 bp fragment of the candidate genes of *GhANR2*, *GhLAR1* and *GhCHS2* was amplified as our previous paper [69]. The deficiency of *phytoene desaturase* gene (*PDS*) causes loss of chlorophyll and carotenoids, showed the typical photobleaching phenotype, was used as a positive marker to visualize the timing and extent of endogenous gene silencing. The *PDS* gene fragment was cloned from C312 genomic DNA by PCR to construct vector pCLCrV-*GhPDS* according to the previous paper [69]*.*

The four vectors were transformed individually into *Agrobacterium tumefaciens* strain GV3101 [69]. The pCLCrVA-empty was used for a negative control (N CK), the pCLCrV-*PDS* was used for a positive control (*GhPDSi*), the pCLCrVA-*GhCHS*, pCLCrVA-*GhANR*, pCLCrVA-*GhLAR* and pCLCrVB (for target gene silencing) were used. Plants were transformed with pCLCrV-*PDS* and the pCLCrVA-empty vectors as controls. The primers for cloning and detection in the experiment are listed in Additional file 1: Table S1.

Cotton seedlings were grown in a growth chamber at 28 °C with a 14 h light and 10 h dark cycle. Healthy 2 week old seedlings were infiltrated with different *Agrobacteria* carrying pCLCrVA or one of its derivatives and pCLCrVB. *Agrobacteria* harboring pCLCrVA or one of its derivatives was mixed with an equal volume of *Agrobacteria* harboring pCLCrVB. The mixed *Agrobacteria* solutions were infiltrated into the abaxial side of the cotyledons of the 2-week-old cotton seedlings using syringes without needles. The agroinfiltration was carried out at least three times with at least 30 plants for each vector. DNA was isolated from the leaves of pCLCrV-inoculated cotton plants. The infected plants was detected by PCR using specific primers of CLCrVA F and CLCrVA R [69]. The wild-type C312 and ZX1 or HZ or ZH were used as controls in the experiment. The leaves and developing bolls in transgenic *GhCHSi*, *GhANRi*, *GhLARi*, *GhPDSi* and CKs plants were respectively collected at 0 DPA, 5 DPA, 10 DPA and 15 DPA for measurement of anthocyanin content and gene expression analysis.

### Gene expression analysis by quantitative real-time PCR

Total RNA was isolated from the leaves, the mixture of fiber and seedcoat according to the manufacturer’s instructions (RNAprep Pure TIANGEN BIOTECH, China), and treated extensively with RNase-free DNase I. Double-stranded cDNA was synthesized from 200 ng RNA using FastQuant RT kit with gDNase (TIANGEN BIOTECH, China) according to a standard double-stranded cDNA synthesis protocol. Real-time PCR (qRT-PCR) assays were performed using the SYBR FAST qPCR kit (KAPA SYBR®, USA) and the qRT-PCR reaction was performed using the ABI QS3 fluorescence quantitative PCR instrument (ABI, USA). The PCR amplification system and program were as described previously [69], three biological replications were performed with independently isolated RNA in all the qRT-PCR assays. Relative gene expression levels were calculated using the 2^–ΔΔCt^ method [69]. The expression of *GhANR* genes, *GhLAR* genes and *GhCHS* genes were standardized to the constitutive *GhUBQ7* gene expression level (cotton *Ubiquitin7* gene, Gen Bank accession number: DQ116441, used as reference gene). The primers are listed in Additional file 1: Table S1.

### The analysis of anthocyanin content of transgenic plants

Measurements of anthocyanidin accumulation were performed as described previously [71, 72]. Weighed samples (approx. 100 mg) in a 1.5 mL microfuge tubes were harvested into liquid nitrogen to freeze plant tissue. Samples were extracted overnight in 1 ml of 0.5% (v/v) HCl in methanol, and then violently shaken in vortex for 30 s. The extraction buffers were shaken with 120 rpm in the dark for 1 h. The extraction buffers were centrifuged at 2630 g for 15 min at 20 °C. This process was repeated 3 times. The supernatant was assayed spectrophotometrically (UV-2600, Shimadzu, Japan) and anthocyanidin absorbance units (A_530_–A_657_) per gram fresh weight were calculated. The blank should be 480 ml Methanol with 0.5% (v/v) HCl and 320 ml Milli-Q H_2_0 for a total of 800 ml. A spectrophotometer was used for the absorbance measurements at 530, 620, and 650 nm. The results were determined based on the following equation: optical density (OD) = (A_530_ – A_620_) – [0.1 × (A_650_ – A_620_)] [73].

### Statistical analysis

All data are presented as mean ± SD from at least three independent experiments with three replicates each. The statistical significance of the differences was determined using the Student’s t-test. Differences between treatments were considered significant when *P < 0.05, **P < 0.01 and ***P < 0.005 in a two-tailed analysis.

## Supplementary information

**Additional file 1: Table S1.** Primers used in the experiments. **Figure S1.** Relative expression levels of *GhCHS*, *GhANR*, *GhANR* in the developing fiber of 5 DPA, 10 DPA and 15 DPA in *GhCHSi*, *GhANRi*, *GhANRi*, *GhPDSi* transgenic cotton lines and wild cotton ZX1.

## Data Availability

All data generated or analyzed during this study are included in this published article.

## Abbreviations

ANR: Anthocyanidin reductase
CHS: Chalcone synthase
CLCrV: geminivirus Cotton leaf crumple virus
DPA: Days post anthesis
LAR: Leucoanthocyanidin reductase
PA: Proanthocyanidins
PDS: Phytoene desaturase
UF3GT: UDP-glycose: flavonoid-3-O-glycosyltransferase
