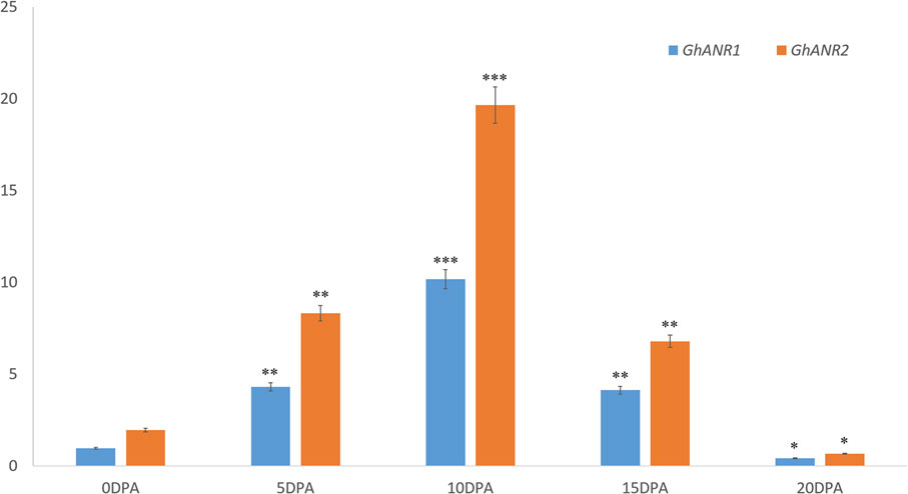# Correction to: Functional analysis of *GhCHS*, *GhANR* and *GhLAR* in colored fiber formation of *Gossypium hirsutum* L

**DOI:** 10.1186/s12870-019-2211-2

**Published:** 2020-01-19

**Authors:** Jianfang Gao, Li Shen, Jingli Yuan, Hongli Zheng, Quansheng Su, Weiguang Yang, Liqing Zhang, Vitalis Ekene Nnaemeka, Jie Sun, Liping Ke, Yuqiang Sun

**Affiliations:** 1 Plant Genomics & Molecular Improvement of Colored Fiber Lab, Key Laboratory of Plant Secondary Metabolism and Regulation of Zhejiang Province, College of Life Sciences and Medicine, Zhejiang Sci-Tech University, Hangzhou, 310016 Zhejiang China; 2 College of Agriculture/The Key Laboratory of Oasis Eco-Agriculture, Shihezi University, Shihezi, 832000 Xinjiang China

**Correction to: BMC Plant Biol (2019) 19:455**

**https://doi.org/10.1186/s12870-019-2065-7**

In the original publication of this article [1], the authors pointed out the Fig. 4b was same with Fig. 4c. The correct Fig. 4b should be below.

The publisher apologizes to the readers and authors for the inconvenience.

**Figure:**