# Supplementary material for: Functional analysis of GhCHS, GhANR and GhLAR in colored fiber formation of Gossypium hirsutum L
Source: BMC Plant Biol. 2019 Oct 29;19:455. doi: 10.1186/s12870-019-2065-7 (PMC6819470; doi:10.1186/s12870-019-2065-7)
Supplement: Supplementary file 1 — Additional file 1: Table S1. Primers used in the experiments. Figure S1. Relative expression levels of GhCHS, GhANR, GhANR in the developing fiber of 5 DPA, 10 DPA and 15 DPA in GhCHSi, GhANRi, GhANRi, GhPDSi transgenic cotton lines and wild cotton ZX1. [file 12870_2019_2065_MOESM1_ESM.docx]

Additional files:

Table S1 Primers used in the experiments

| Primer use | GENE ID | Primer F/R | | Consequence |
| --- | --- | --- | --- | --- |
| RT-qPCR | LOC107938221 | | *ANR1-F* | GTGCTGTCAATACCAGTGTGC |
|  |  | | *ANR1-R* | CGATCCCATACTTAAAGCTGAATCC |
| RT-qPCR | LOC107905961 | | *ANR2-F* | GACGTTGGCTGAAAAGGCAG |
|  |  | | *ANR2-R* | CAGAAAAACATGGGCTCGGC |
| RT-qPCR | LOC 107946451 | | *LAR1-F* | TCTGCCGGCACAGTTAAG |
|  |  | | *LAR2-R* | TTGACGCTGCCATCTCCATA |
| RT-qPCR | LOC 107931042 | | *LAR2-F* | GCATCTTGGACCAGCTCAGT |
|  |  | | *LAR2-R* | GTGTTGTCATGGTAGGGCCA |
| RT-qPCR | LOC 107934794 | | *LAR3-F* | ACTCTGTCGGCACTGTTAAG |
|  |  | | *LAR3-R* | AAGGGTGGAATGACCTCGGA |
| RT-qPCR | LOC107927759 | | *CHS-1-F* | GGTGCCGATTACCAGCTCAC |
|  | LOC107957085 | | *CHS-1-R* | CACACGGAGCACAGTACCAC |
| RT-qPCR | LOC107888447  LOC107888463  LOC107897841  LOC107897842  LOC107914891  LOC107914895  LOC107912380 | | *CHS-2-F* | GCTGTTACCTTTCGTGGACC |
|  |  | | *CHS-2-R* | TCGCACCATCACTATCTGGC |
| RT-qPCR | LOC107914136 | | *CHS-3-F* | GCAAATCCGCCCCATTGTTT |
|  |  | | *CHS-3-R* | ATCTTCCGTCAGGTGCATGT |
| RT-qPCR | DQ116441 | | *UBQ7-F* | GAAGGCATTCCACCTGACCAAC |
| Reference gene |  | | *UBQ7-R* | CTTGACCTTCTTCTTCTTGTGCTTG |
| *GhANR* RNAi | LOC107905961 | | *V ANR-F* | *GG*ACTAGTGCTTGTGTCGTGGGTGGCA |
|  |  | | *VANR-R* | *TT*GGCGCGCCTGCGGCAGATGATGTCAAGA |
| *GhLAR* RNAi | LOC107931042 | | *V LAR-F* | *GG*ACTAGTCCAACGTATATCTTAGTCCGCTCT |
|  |  | | *V LAR-R* | *TT*GGCGCGCCCTCCTAATCTTGCTCTTTTGCT |
| *GhCHS* RNAi | LOC107897841 | | *V-CHS-F* | *GG*ACTAGTGCACAAAACCGAGTTGAAAGA |
|  |  | | *V-CHS-R* | *TT*GGCGCGCCGGTCCACGAAAGGTAACAGCA |
| VIGS | *GhPDS* RNAi | | *V-PDS-F* | *GGAC*TAGTGCCTGAAGACTGGAGAGAGATTT |
|  |  | | *V-PDS-R* | *TTGG*CGCGCCGCTTTACTCTGATCCGCAGATATT |
| Carrier detection |  | | *A-F* | ATTTTGCGCCTGACTAGCCT |
|  |  | | *A-R* | CGAATTTTCAACGTTGCATACA |

Italic sections are protective bases, and underlined sequences are restriction enzyme sites.

Figure S1 Relative expression levels of *GhCHS*, *GhANR*, *GhANR* in the developing fiber of 5 DPA, 10 DPA and 15 DPA in *GhCHSi*, *GhANRi*, *GhANRi*, *GhPDSi* transgenic cotton lines and wild cotton ZX1.

A: Relative expression analysis of *GhLAR* in *GhCHSi*, *GhPDSi* transgenic cotton lines and WT.

B: Relative expression analysis of *GhANR* in *GhCHSi*, *GhPDSi* transgenic cotton lines and WT.

C: Relative expression analysis of *GhCHS* in *GhANRi*, *GhPDSi* transgenic cotton lines and WT.

D: Relative expression analysis of *GhLAR* in *GhANRi*, *GhPDSi* transgenic cotton lines and WT.

E: Relative expression analysis of *GhCHS* in *GhLARi*, *GhPDSi* transgenic cotton lines and WT.

F: Relative expression analysis of *GhANR* in *GhLARi*, *GhPDSi* transgenic cotton lines and WT.

Data presented in all graphs are means±SD (n =3), (Student’s t-test, *P < 0.05, **P < 0.01, ***P < 0.005, compared to WT)

.


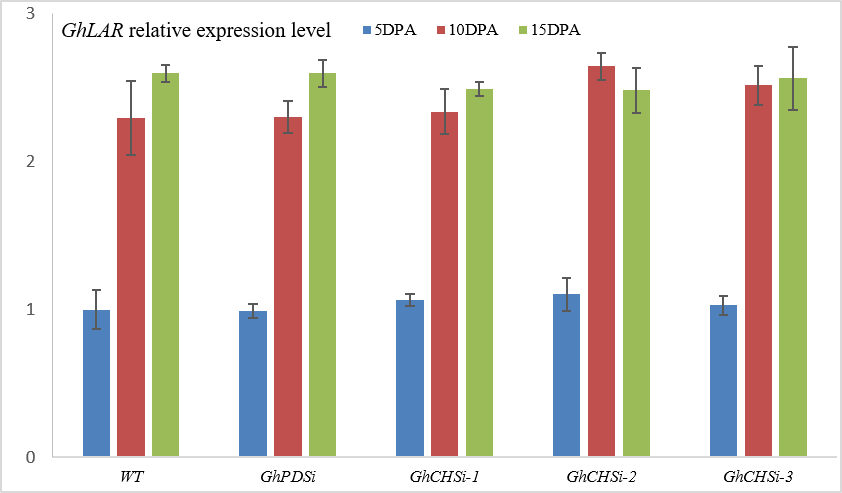


Figure S1A: Relative expression analysis of *GhLAR* in *GhCHSi*, *GhPDSi* transgenic cotton lines and WT.


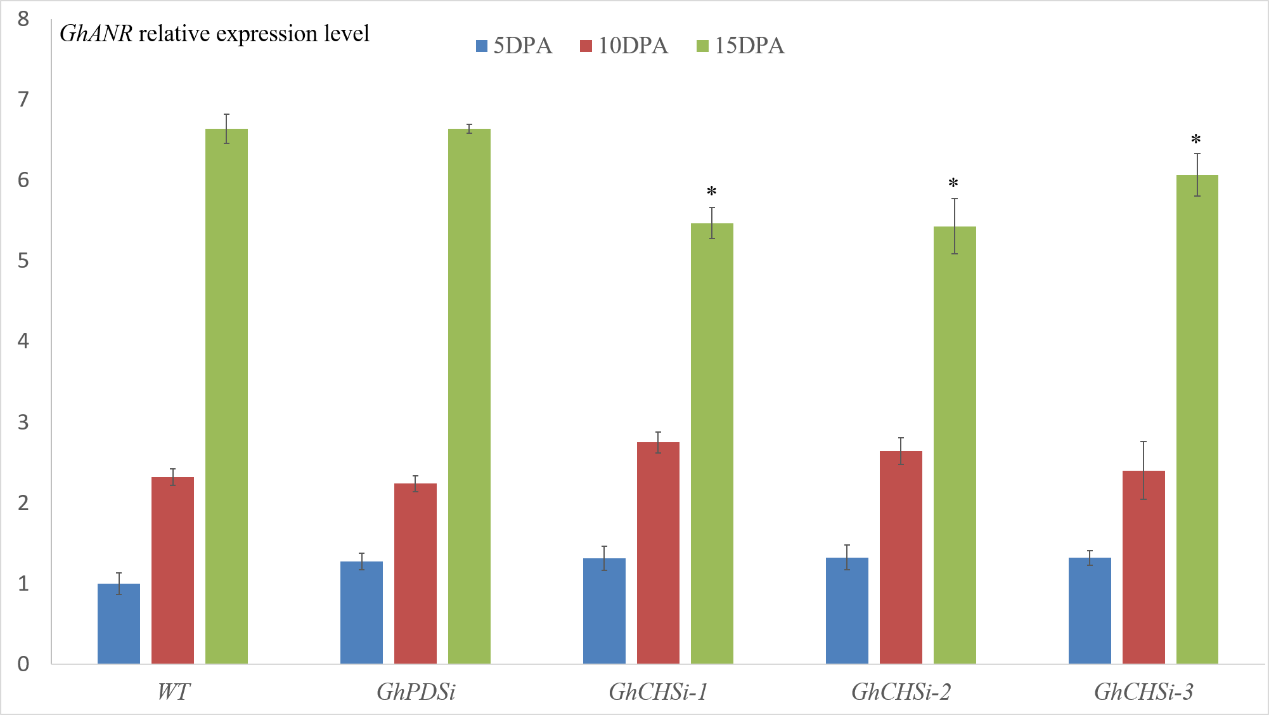


Figure S1B: Relative expression analysis of *GhANR* in *GhCHSi*, *GhPDSi* transgenic cotton lines and WT.


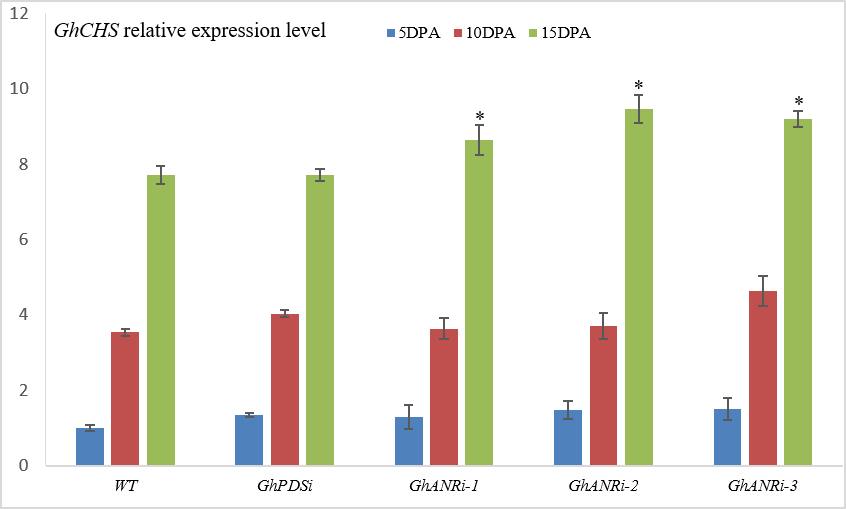


Figure S1C: Relative expression analysis of *GhCHS* in *GhANRi*, *GhPDSi* transgenic cotton lines and WT.


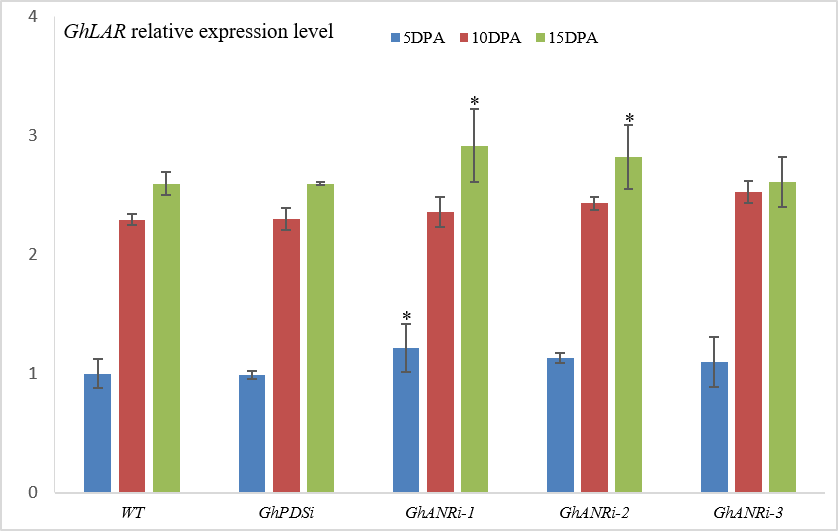


Figure S1D: Relative expression analysis of *GhLAR* in *GhANRi*, *GhPDSi* transgenic cotton lines and WT.


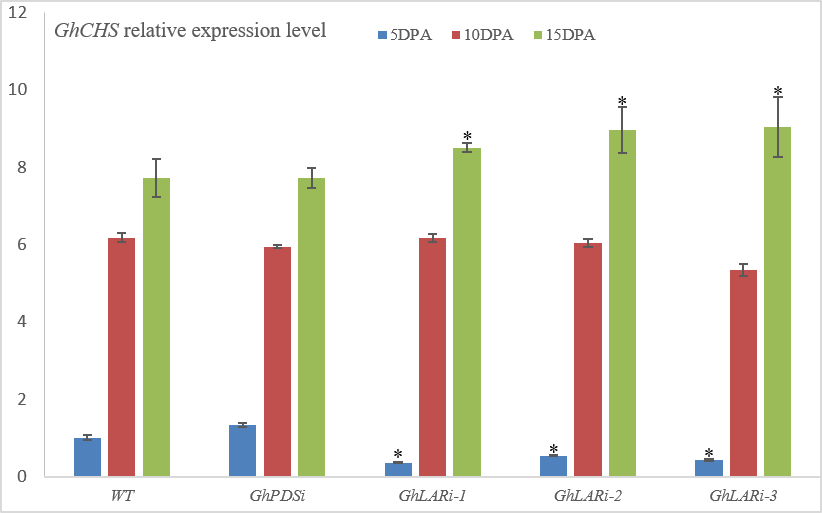


Figure S1E: Relative expression analysis of *GhCHS* in *GhLARi*, *GhPDSi* transgenic cotton lines and WT.


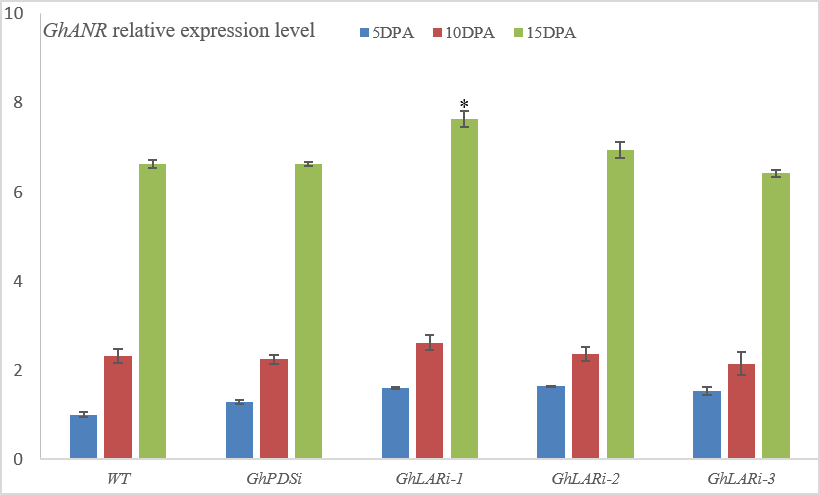


Figure S1F: Relative expression analysis of *GhANR* in *GhLARi*, *GhPDSi* transgenic cotton lines and WT.
